# A Neural Networks-Based Hybrid Routing Protocol for Wireless Mesh Networks

**DOI:** 10.3390/s120607548

**Published:** 2012-06-07

**Authors:** Nenad Kojić, Irini Reljin, Branimir Reljin

**Affiliations:** 1 Digital Image Processing, Telemedicine and Multimedia Laboratory, Faculty of Electrical Engineering, University of Belgrade, Bulevar Kralja Aleksandra 73, Belgrade 11000, Serbia; E-Mails: irinitms@gmail.com (I.R.); reljinb@etf.rs (B.R.); 2 ICT College for Vocational Studies, Zdravka Čelara 16, Belgrade 11000, Serbia; 3 Faculty of Electrical Engineering, University of Belgrade, Bulevar Kralja Aleksandra 73, Belgrade 11000, Serbia; 4 Innovation Center of Faculty of Electrical Engineering, University of Belgrade, Bulevar Kralja Aleksandra 73, Belgrade 11000, Serbia

**Keywords:** artificial intelligence, Hopfield neural network, MANET, multicriteria optimization, routing protocol, wireless mesh network

## Abstract

The networking infrastructure of wireless mesh networks (WMNs) is decentralized and relatively simple, but they can display reliable functioning performance while having good redundancy. WMNs provide Internet access for fixed and mobile wireless devices. Both in urban and rural areas they provide users with high-bandwidth networks over a specific coverage area. The main problems affecting these networks are changes in network topology and link quality. In order to provide regular functioning, the routing protocol has the main influence in WMN implementations. In this paper we suggest a new routing protocol for WMN, based on good results of a proactive and reactive routing protocol, and for that reason it can be classified as a hybrid routing protocol. The proposed solution should avoid flooding and creating the new routing metric. We suggest the use of artificial logic—*i.e.*, neural networks (NNs). This protocol is based on mobile agent technologies controlled by a Hopfield neural network. In addition to this, our new routing metric is based on multicriteria optimization in order to minimize delay and blocking probability (rejected packets or their retransmission). The routing protocol observes real network parameters and real network environments. As a result of artificial logic intelligence, the proposed routing protocol should maximize usage of network resources and optimize network performance.

## 1. Introduction

Modern information society has the increasing need for Internet access, as the global network for information circulation. Nowadays, it is necessary to obtain Internet access for all types of network devices (fixed and mobile wireless devices), in any possible way and at any place. This is extremely important if we bear in mind that the use of wireless devices, like smart phones, mobile phones, PDAs, laptops *etc.* is on the increase. These demands impose the need for large infrastructure, which is often impossible to realize, especially in rural areas.

**Figure 1.**
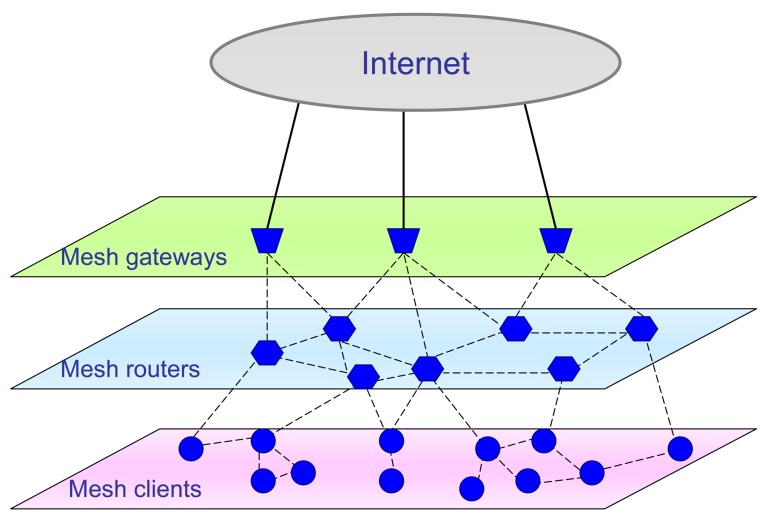
The structure of the WMN.

This type of problems can be solved to a great extent with the use of Mobile *Ad Hoc* Networks (MANETs) [1–5]. MANET presents large-scale multi-hop wireless network which is very applicable for quick access and wide area coverage [1,3,5]. One specific type of mobile *Ad Hoc* network is the wireless mesh network (WMN) [6–9]. A WMN is the set of wireless nodes, which communicate in such a way that they mutually transfer all packets within the network. This relatively new wireless multihop technology is organized as a mesh topology and can be a revolutionary solution for gaining access to wireless networks [10]. Like in a MANET organization, a WMN also has nodes that behave simultaneously both as host and as wireless router. In that way, each node has both mesh router and mesh client functions. The most common role of WMNs is to gain quick wireless network access and connection to the Internet. For that reason, there are two main types of nodes: the nodes that are part of the network and don't have direct connection to Internet (called WMN routers), and nodes that do have Internet connection (called gateways) [11]. There is a distinction between several types of links within the network, where only intra-mesh links are wireless and all others can be either wired or wireless. As an illustrative example, in Figure 1 the block diagram of typical WMN is depicted.

Link connections between gateways and Internet can also be wired or wireless and are is supposed to be reliable, so it will not be discussed in the paper further on. A WMN defined in this way is suitable for establishing wireless communication between subscribers and the Internet. Standard new generation devices (laptops, PDAs, PocketPCs, phones, *etc.*) which have wireless network interface cards (NICs) can be directly connected on wireless mesh routers, and that way access the Internet through a WMN. Subscribers who don't have NIC can be connected to the WMN using Ethernet directly through mesh routers.

WMN defined in this way is dynamically self-organized, self-configured, self-optimizing and fault tolerant with the nodes in the network automatically establishing and maintaining mesh connectivity among themselves [12,13]. All these performances clearly indicate that WMN has advantages such as low investment overhead, easy network maintenance, fast to deploy, robustness and reliable service coverage. In that way, WMNs have become a favorable solution for neighborhood networks, enterprise networking, communities, *etc.* A large number of companies have based their solutions on WMNs. IP solutions using WMNs have been also created by Nokia, Microsoft, Motorola, Intel, *etc.* [11,12].

Constructing WMNs requires initial creation of a network from various numbers of mesh routers and mesh clients. Mesh routers can be stationary, set on the building roofs or high spots in the air, while mesh clients can be mobile and in that way communicate with the other wireless routers.

The gateways are mostly stationary wireless routers, which make connections with other networks or Internet through a wired medium. In this way, the price of Internet access has decreased dramatically compared to classical wired systems (cable-modem and xDSL). So, the advantages compared to fixed wireless metropolitan area networks (WMANs) are seen in multiplied routes. This comes from using mesh clients as routers, thus compensating for the bad performance of some space directions; bad flow or connection failures produced by obstacles (buildings, trees, *etc.*). Further, for implementing WMNs, already available components, coming from other technologies, can be used such as: *Ad Hoc* network routing protocols [5,14,15], IEEE 802.11 MAC protocol, wired equivalent privacy (WEP) security, *etc.* Unfortunately, these solutions can offer some but not all of the necessary performance needed to satisfy final subscribers. As one of the main problems, optimal routing which considers all important parameters of WNM topology stands out [14,15].

The advantages of WMNs are: (1) Reliability—the ability of the protocol to fulfill quick rerouting in the case of link failure between nodes in overall route; mobile user connectivity—protocol should enable fast hand-offs; (2) Scalability: in the case of overloading, the number of nodes and QoS in the network cannot be expanded, so various categories of traffic should be delivered to the optimal routes which can satisfy needed quality of service [11].

On the other hand, wireless routers in WMNs are expected to be unstable. Apart from routers, wireless links can be unstable. The quality of data transmission can be weakened by multi-path fading effects, external interference and weather conditions. The potential instability of routers and links can appear after determining the optimal route, during transmission of data, which brings us additional requirements related to the protocol.

Existing routing protocols applied to WMNs do not have enough scalability [14,16]. Some protocols perform an additional check after finding an optimal route, but it is certain that the conditions can changed over time [16]. Usually, the changes are detected only when the link fails or if some of the routers do not respond [17,18]. The authors of [19,20] suggested some solutions, where the route testing is done periodically, but the testing interval is not small enough to follow current network monitoring. Besides, the protocol should obtain adaptability in a sense of topology change, as well as adaptability of routers and links, which is quite often in WMNs. In addition to the problem of defining the optimal routing protocol, while designing the WMN, the problem of the quality of wireless transmissions has to be considered. Several authors have provided suggestions for solving the transmission problem and making optimal use of the available spectrum [21–23].

In this paper, one solution for optimal routing in WMNs is proposed, more specifically, the one emphasizing the management of network resources. Taking into consideration all particularities of WMNs, the proposed solution provides, in the Pareto sense, the optimal route selection, in the shortest time interval, taking into account the current state of the network and optimal use of all network resources. In that way, it is necessary to choose the route in accordance with the needs of future subscribers and their traffic, with as small as possible number of rerouting and the optimal loading balance of the entire network.

The rest of this paper is organized as follows. The brief view of related work is given in Section 2. Section 3 describes the methodology including network deployments and protocol description. The used artificial neural network model is explained in Section 4, while new routing metric based on Hopfield neural network is given in Section 5. Section 6 provides our simulation results. At the end we summarize our work and give future plans in the Conclusions.

## 2. Related Work

A number of different routing protocols have been presented in the literature. Some were used many years ago, and had been implemented in wired networks [11,14,17,24,25]. Starting with the Routing Information Protocol (RIP) [26] and its modification [27] there is Open Shortest Path First (OSPF) [24,25,28] as an open protocol. OSPF is one of the most used Interior Gateway Protocols (IGP) in large enterprise networks. This protocol is an adaptive routing protocol, which means the routing is based on real network parameters. The algorithm proposed in this paper will use some of the OSPF attributes, improving the mechanism of collecting, processing and routing decisions. In large networks the Intermediate System to Intermediate System (IS-IS) is commonly used, as one of link state protocols [29]. The next step after interior gateway protocol implementation is the exterior gateway protocol. Border Gateway Protocol (BGP) is one of the most used protocols for routing information between autonomous systems on the Internet [30]. All these protocols were initially developed for wired networks, but the main logic can be implemented in wireless routing protocols. One of the major problems is stability of the wireless links. This characteristic could produce a problem related to the quality of service as well as the possibility to guarantee services [25,31].

With regard to the wireless routing protocols, the most important solutions are aimed for *Ad Hoc* networks [32–34], addressing table-driven protocols as well as possibility for MANET implementation. Most popular on-demand routing protocols are opposite to table-driven routing protocols. Highly dynamic topology changes, users' connection and traffic density need new protocols especially designed for *Ad Hoc* networks. *Ad Hoc* On Demand Distance Vector (AODV) and Dynamic Source Routing (DSR) are the most used solutions for these purposes [18,35]. A lot of authors have offered some improvements for these protocols, trying to optimize networks resources [36,37].

In some papers, the focus is based on node location information in order to reduce routing overhead [38,39]. The weakness of each routing protocol is addressed to exciding nodes number, exciding traffic density and network mobility. So, several papers have analyzed the performance of network scalability [40,41] in order to realize better performances in large networks. The Optimized Link State Routing Protocol (OLSR) [42] is a specifically created protocol based on a well-known link state protocol, and optimized for better network features and resource utilization.

Extensions to existing routing protocols, AODV [18], DSR [35] and Simple Opportunistic Adaptive Routing Protocol (SOAR), are presented in [43]. Besides, existing protocols in a hierarchical, two-level MANET, are proposed in [44]. One solution of Hybrid-AODV addressed to packet delivery ratio and delay for large networks is also proposed. Our algorithm is going to optimize packet delivery ratio and in the same time, produce as good as possible load balancing. One fast protocol for small networks is shown in [42]. The reduction of three hops is too small for larger networks and we use it in our work. The implementation for larger network is given in K-hops routing protocol [45]. The authors offer an extension of AODV with at most K hops, but contrary to our solution, they use the flooding mechanism. A protocol for using multiple routes at short time-scales is given in [46,47]. Some improvements of AODV based on QoS are given in [48].

Hybrid routing protocol addressed at WMNs is given in [49–52]. This protocol is based on proactive tree formations with periodic network flooding. However, we do not use flooding mechanism in our protocol.

Some improvements of network performance by multiple radios and channels are given in [20,53–55]. The authors suggest a fully distributed channel assignment algorithm for dynamic traffic adoption. This algorithm assumes a static wireless mesh network that is different from our dynamic network topologies. Also, this algorithm is based on multi-radio or multi-channel routing, opposite to our assumptions.

A lot of unicast routing protocols [18,35,56] and some improvements [57] are related to MANETs. Some of their extensions with gateway discovery are given in [58–62]. Most of these protocols use hop-by-hop routing, that is completely different from our routing protocol based on multicriteria optimization and a centralized approach.

In recent years, more research has focused on the development of dynamic and scalable protocol in WMNs, especially analyzing density [63]. This is very important if we use WMN for multimedia streaming [64].

Some modifications of protocols are caused by *Ad-Hoc* 802.11s-based networks [65,66]. In accordance with the application of protocols in mobile devices, and various types of applications, some research was to develop multi path protocols [67,68]. At the same time, several papers describe cross-layer based proactive routing protocols [69,70]. Several improvements for AODV based on process algebra are given in [71].

As a concluding remark, existing routing protocols use flooding in order to discover and maintain routes between users and gateways within MANETs. In WMNs, most of the traffic is assumed to flow to and from the gateways connected to the Internet [11]. Based on that, we have designed a new routing protocol without flooding. Furthermore, we propose a new routing metric based on artificial logic and optimal multicriteria optimization.

## 3. Methodology

The routing algorithm proposed in this paper is based on link state routing protocols [10,11,24,28,44]. Considering the number of hops, bandwidth, load and delay we have created a new metric. This metric uses artificial neural network logic in order to optimize route selection, optimize network resources and relax link occupancy. In this way, the probability of network blocking should be minimized. The proposed routing algorithm is based on two different Hopfield neural networks. The first one is dedicated to mobile agent routing logic, in order to ensure that every change of network topology or network parameters is distributed to the network as fast as possible [72–74]. The other one is dedicated to the routing protocol and the route selection problem based on previously collected information. All these artificial logics are implemented in our algorithm bearing in mind that WMN's attributes (dynamic topologies changes, route stability, and relation between bandwidth and load) need very specific logic and good potential redundancies.

### 3.1. Network Deployments

**Figure 2.**
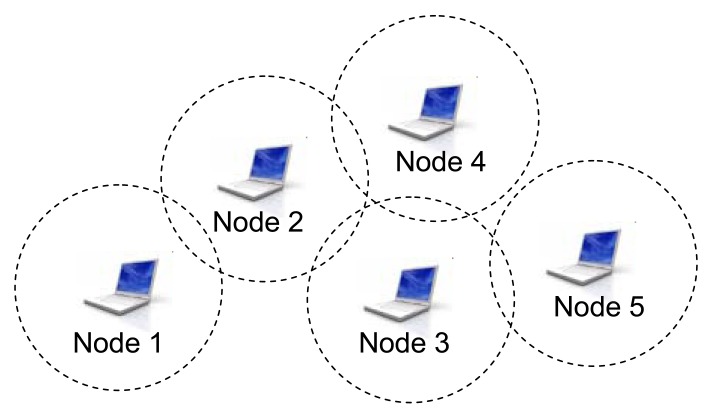
One example of a WMN consisting of five nodes.

The first step in our algorithm is the mathematical representation of the network topology. Furthermore, our model is able to generate and operate with arbitrary number of WMN routers (nodes). Every node can be connected to an arbitrary number of nodes. Two nodes are connected by a link which can be described by several parameters (cost, bandwidth, load, delay, *etc.*). Our model assumes the possibility of bidirectional links meaning that one link has different parameters for different directions. In this way, the network parameter should be similar as much as possible to the real network environment. Let's imagine that we have five nodes with their wireless access (marked as circles), as shown in Figure 2.

**Figure 3.**
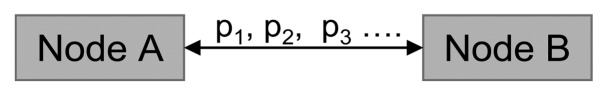
Representation of symmetrical link.

Every two or more nodes with spatial overlapping are detected as directly connected nodes. In that way the nodes connectivity and network topology are defined. As mentioned before, several link parameters *p*_1_, *p*_2_,…, *p_M_* are assigned to every link. If these parameters are symmetrical for any direction, the network can be represented as shown in Figure 3.

**Figure 4.**
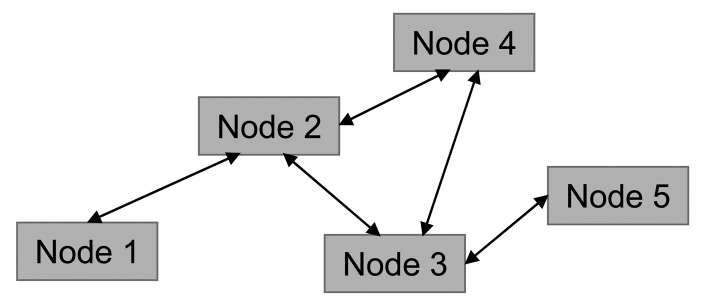
Oriented graph as network representation.

In this way the network topology (shown in Figure 2) can be represented as an oriented graph (Figure 4).

**Figure 5.**
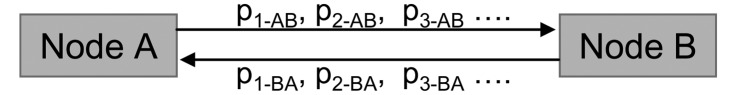
Representation of bidirectional link.

**Figure 6.**
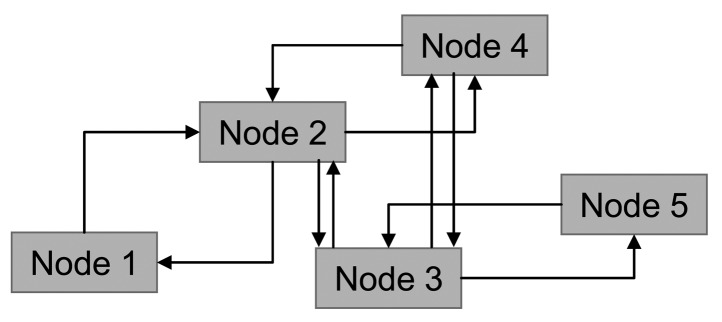
Oriented graph as network representation with bidirectional link.

In our case, where network links are bidirectional, as shown in Figure 5, the network topology is given in Figure 6.

Our model automatically creates a mathematically oriented graph and makes two types of matrices: *connectivity matrix* and the *weight matrix*. All matrices are of the dimension *N* × *N*, where *N* is the number of nodes. The connectivity matrix has the value of 1 on the position *i*, *j* if node *i* is directly connected to node *j*. Otherwise, the matrix element is 0.

There can be one or more weight matrices. The value of weight matrix on position *i-j* is determined by the value of appropriate parameter on link connecting nodes *i* and *j*. In this way, after network representation (or network exploration) we have one connectivity matrix and *M* weight matrices for every of *p_M_* link parameters.

### 3.2. Protocol Description

As mentioned before, routing is one of the most important and the most critical parts for regular operation of a WMN. The traffic in a WMN is mostly based on communication between the user and the Internet. Significantly minor traffic is addressed to internal user communication. From that reason the route selection problem is oriented on finding the optimal route between a user and a gateway. Besides that, the routing protocol should provide efficient and fast detection of changes in the network topology, as well as changes of link parameters and potential connections of new users. Generally, routing protocols in an *Ad Hoc* network could be classified into three categories [3]:

1. *Proactive* (table driven) routing protocols,
2. *Reactive* (on demand) routing protocols, and
3. *Hybrid* routing protocols.

Proactive routing protocols are based on continuous information refreshing in routing tables. Information on any change in the network is sent at constant time periods. The main goal is to maintain up-to-date information in routing tables thus enabling the route selection in a most excellent manner. Some of the representative protocols based on table driven logic are OLSR and Destination sequenced distance vector (DSDV) [3,17].

Counting to infinity and maintaining up to date information may represent very difficult problems [9,11]. One solution could be obtained through adding a special number to every node which will be incremented every time when a node environment change occurs, so a node with a higher number indicates that the node has refreshed information about changes. Based on this refreshed information, the route should be calculated again. The new route is stored in a local routing table. On the other hand, exchanges of the routing tables often have influence on network performances and increase time of convergence. The additional problem could be the size of routing table especially if a node number increases during time. In that case, all information cannot be stored in one Network Protocol Data Unit (NPDU) [3]. It should be pointed out that asynchronous exchanging of routing table information indicates dump routing fluctuation. A solution for this problem demands rationalization in new path selection, especially if it is known that new routing information is coming soon. With new information protocols, the route recalculation should be made.

Reactive routing protocols calculate optimal route on demand. When a route is calculated, it is stored and used until the destination is available or the path's time is out. The mostly used reactive protocols are DSR and AODV [18,35].

In most cases these protocols have only information about next hops in the routing table. This information is time limited and will be automatically deleted if some of information is not used during the time. In this case, a new route calculation demands sending of Route Request (RREQ) packets to the neighborhoods [3,9]. Having received this packet every neighbor sends the same packet to its neighbors. This logic is repeated until the RREQ packet is delivered to the destination node. After that, the destination node should reply by a Routing Reply (RREP) packet.

In order to create a better solution, a hybrid solution gives the possibility to combine some of the advantages of proactive and reactive routing protocols. The routing protocol proposed here is a hybrid routing ones. As a reactive routing protocol, the proposed algorithm should find the optimal path on demand, based on up-to-date information. The reactive routing protocol and the procedure of sending a large number of RREQ and RREP packets are supposed to send information in discrete time intervals, after the routing request. On the contrary, the proposed algorithm should provide continuous updating of the routing information. Updated information should be available at every moment, thus decreasing the possibility of wrong routing decisions. On the other hand, the proposed solution should not send a complete routing table, but just changed parameters. In order to accelerate computational time and routing process, the proposed solution uses previously calculated routes until a new route is established. For the proposed routing protocol realization of the two segments the following are suggested:

1. Optimization of data exchange (all relevant information about network changes),
2. Finding of the optimal multicriteria route in order to use network resource in a best way. Optimal use of network resources is very important from the point of current route selection as same as all future routing in order to minimize blocking probability. In this way, we have realized the network control management as a part of route selection problem.

### 3.3. Mobile Agent Technology

Mobile agent technology offers very good results in quite a large number of implementations [72–74]. Mobile agent is a specific type of distributed software. Its goal is moving through the network and collecting information from the nodes. Leaving a node the mobile agent brings all changes detected in its host routing table. When a mobile agent comes to a new node, it shares the information on the previous nodes with a current routing table. It is obvious that the main problems in mobile agents' implementation are: the moment when an agent starts, its route and number of agents in the network are needed. For the purpose of this routing protocol we suggest using √N agents (where N is the number of nodes). This number is a result of a lot of statistical analyses and represents an empirical value. A larger number of agents would provide faster data exchange but produces more traffic in the network, thus increasing the probability of blocking. √N agents could be an optimal solution for a pretty large number of different network topologies. In the proposed algorithm agents don't have any specific time to start. They are moving through the network all the time, bringing and sharing information. The main problem in agent's usage refers to their moving path. We suggest Hopfield neural network [75] for finding the moving path in similar way as for solving the TSP problem (Traveling Salesman Problem). We propose the shortest path algorithm based on link cost [76] as a metric in the model. Let us assume that cost dedicated to link between node *i* and node *j* is *C_i,j_*. This parameter should be created automatically by modification of bandwidth link parameters *B_i,j_* as:


for (i=1; i<=N; i++)
{
 if (B_i,j_ ∼= 0)
  {C_i,j_ = 0.1;}
 else
{C_i,j_ = 100;}
}

In this way every existing link has the same cost which means that every link has the same probability to be included in the TSP route at position *i*. Taking this initial step, every agent can have a different path generated in a random way. Every agent can access any node at an arbitrary time and share network information with its routing table. It is obvious that the routing algorithm has up to date information independently on the new route demand. Based on the routing table's data, the routing algorithm should find the optimal route between the source and the destination node. This route should be calculated for every single user. Once, when route is found, it will be used for a particular user for a time the connection lasts. If this path, or part of it, becomes unavailable a new route will be found. After finding an optimal route some network resources are going to be occupied for a particular user and information on that (information about the available bandwidth, load and links in use) should be delivered by mobile agents to all nodes. In this way the routing protocol can find an optimal route for every user based on really available network resources, so the network delays, as well as blocking probability (number of rejected packets) will be minimized.

**Figure 7.**
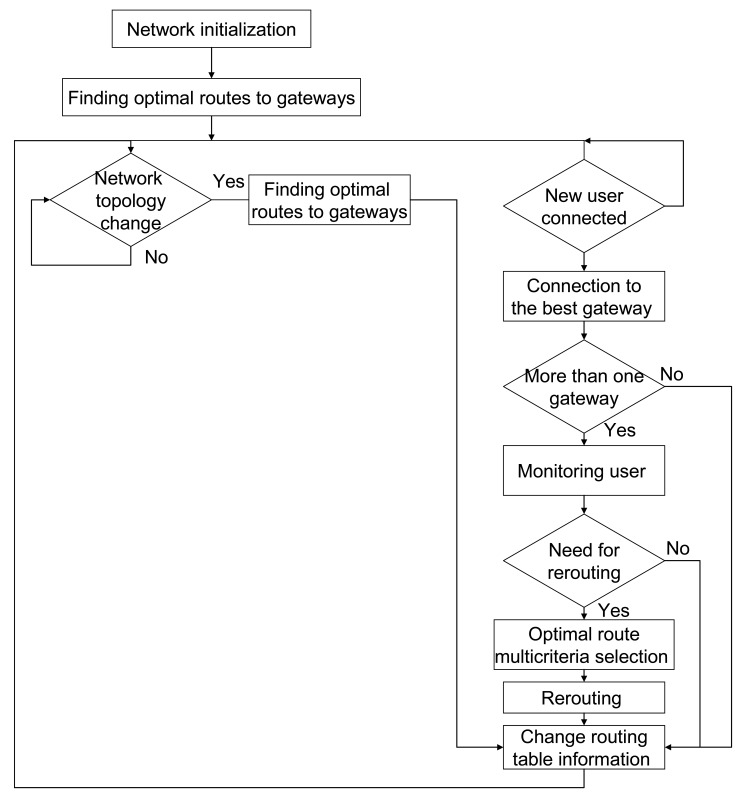
Algorithm of proposed routing protocol.

The proposed algorithm metrics is based on the two essential parameters: bandwidth and load. Two additional parameters: hop numbers and delay, are optional. In this way optimal usage of network resources, as well as possibility for guaranteed QoS should be achieved. The logic of proposed routing protocol is presented by the block scheme in Figure 7.

The main idea is to divide routing protocol into two phases:

- (a) As fast as possible association of a new user,
- (b) Finding the optimal route for a new user, considering its request.

**Figure 8.**
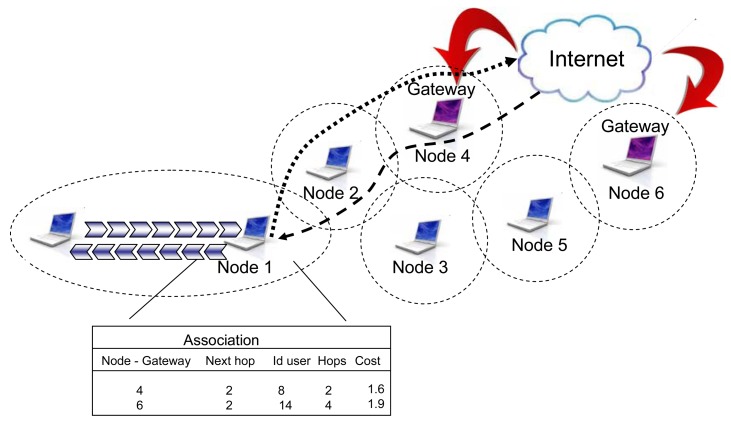
New user association.

The first phase means that, according to the routing table, an optimal route to some of the available gateways has been previously calculated. If a new user wants to make a connection to the network it should send a request to the nearest node in the network (e.g., node 1, Figure 8).

Upon receiving this request, the routing algorithm should read the routing table in node A and find the previously calculated optimal route to the nearest gateway, Figure 8 (next gateway 4 has the minimal hop number 2, so it will be used in this case). The metrics explained in this case are based only on hop count, because this parameter is changing very rarely. The information on the selected route will be send to a new user and after that a connection to the Internet could be established. At the moment of connecting a new user, network topology as well as network parameters are changed. While moving, mobile agents share these pieces of information with all nodes in the network.

In the second phase activation of a parallel process for to the previous one, assigning a route to a new user is done, so the monitoring is addressed to new user's traffic needs and improving of the bandwidth utilization and load parameters on assigned links.

If the artificial logic (Hopfield neural network [76,77]) finds out that some of the links are overloaded, or close to it, or if there is any other alternative solution which makes network downloading, the routing protocol should perform a rerouting. Here, the meaning of “rerouting” is finding of the new route for a new user based on its requirements. As the rerouting is done, monitoring for the user associated is finished. From the network point of view, to perform this change it is necessary to distribute the new information through the network by mobile agents. Taking this information into account, every new user will be associated to the network on the best possible way and with up to date information in its routing table.

**Figure 9.**
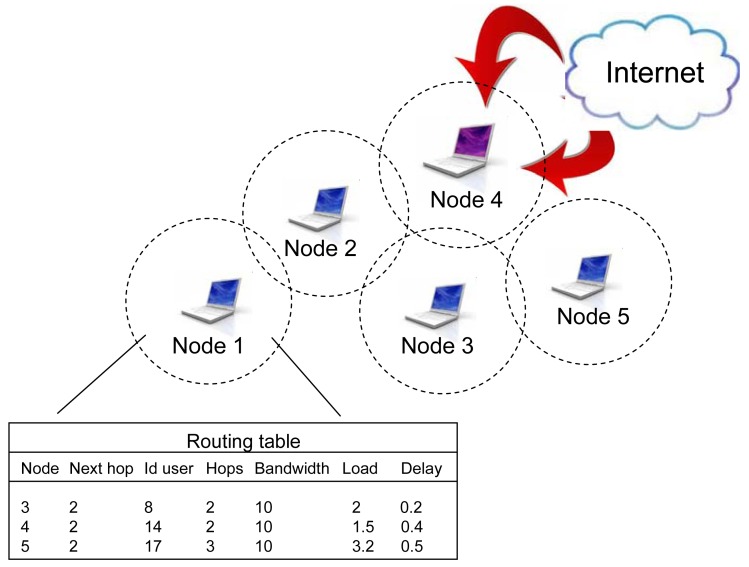
Routing table stored in network's node.

Upon the user's requirements, the traffic should be routed through the network and towards a mesh gateway to Internet, or some of the internal members inside the network. Routing table information takes into account every node in the network. The basic routing table structure for node 1 is represented in Figure 9. Each node represents a minimum one record. Associated data are: Next hop, Total number of hops, Bandwidth of the next hop link, Load of the next hop link, optionally Delay of the next hop link and User identification. If there are more active users for some destinations, an additional record (per every user) will be stored in the table.

## 4. Artificial Neural Networks

Artificial neural networks are formed as a result of comprehensive research and analysis of the human brain and neuron structure. Artificial networks have rapidly found wide applications in different areas where complex relationships, large number of parameters and complicated computer calculations are necessary. As such, neural networks have found themselves as a tool in solving the various problems related to pattern recognition, optimization, data control, coding, Analog-to-Digital conversion, Job scheduling problems, Quadratic assignment and other Nondeterministic Polynomial Time Complete Problems (NP-complete problems), *etc.* [78,79].

### 4.1. Hopfield Neural Network

The Hopfield neural network consists of a set of *N* interconnected neurons which update their activation values asynchronously and independently of other neurons. All neurons are both input and output neurons. The Hopfield neural network is classified as a recurrent neural network and could be represented as content-addressable memory systems with binary threshold units [80].

The conventional Hopfield model is the most commonly used model for auto-association and optimization. Hopfield networks are auto-associators in which node values are iteratively updated based on local computation principle: the new state of each node depends only on its net weighted input at a given time [81].

**Figure 10.**
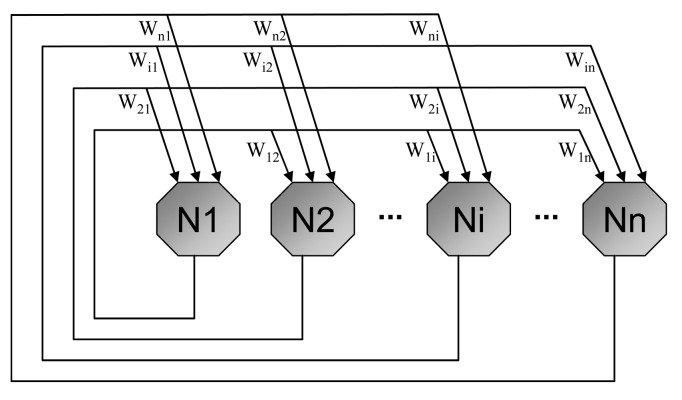
Hopfield neural network architecture.

This network is a fully connected network and the weight matrix determination is one of the important tasks while using it for any application. It is a recurrent single layer and unsupervised network. The architecture of Hopfield neural network is shown in Figure 10.

**Figure 11.**
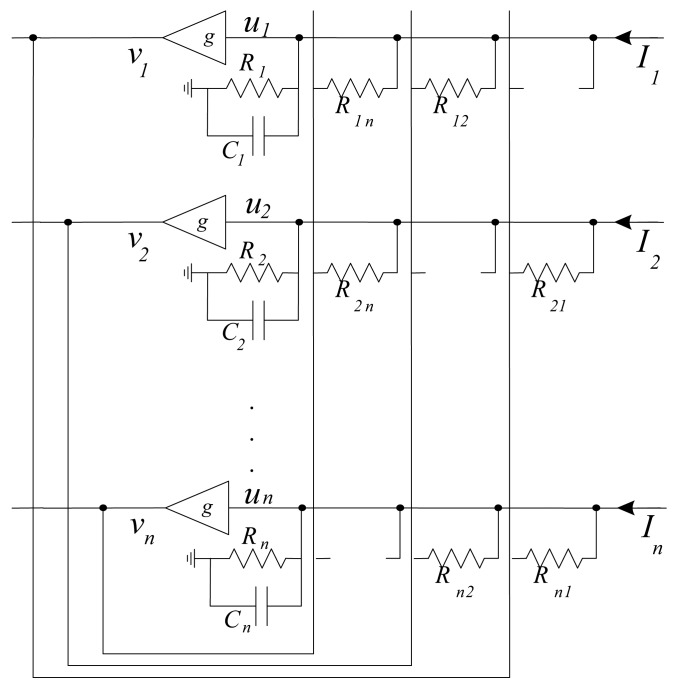
One possible hardware realization of Hopfield neural network.

Each neuron has two states similar to those of McCulloch and Pitts [82], 1 as activation and 0 as deactivation. Each neuron is connected to other neuron by the connectionist weight *W_i,j_*. The model proposed in [83], assumes that there is no self connection, *W_i,i_* = 0, and weights are symmetrical, *W_i,j_* = *W_j,i_*. Possible realization of basic Hopfield neural network model is shown in Figure 11.

**Figure 12.**
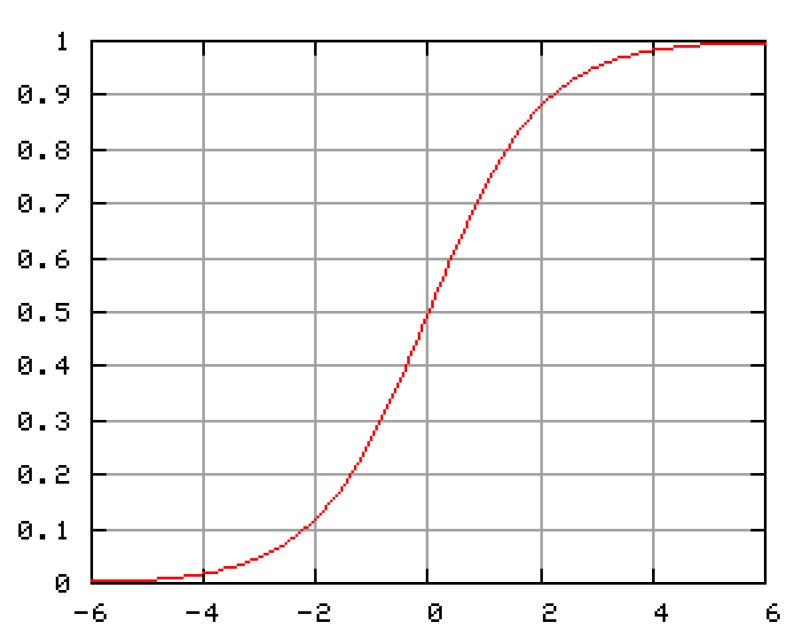
Activation function *v_i_* = *g*(*u_i_*).

Each neuron is realized as a separate block with nonlinear transfer function *g*(*u_i_*) = *v_i_* = *u_i_*, called also an activation function. The most used activation function is the sigmoid one, Figure 12.

(1) $$v_{i}=g_{i}(u_{i})=\frac{1}{1+e^{-a_{i}\cdot u_{i}}}$$

The outputs of each neuron will be fed back to all other neurons through the connection weight matrix **W** = [*W_ij_*]. In hardware realization weight *W_ij_* is related to conductances. One possible hardware realization of Hopfield neural network is shown in Figure 11. Weight *W_ij_* are realized as resistances *R_ij_*, where *W_ij_* = 1/*R_ij_*.

The basic HNN model has two types of inputs: external inputs *I_i_* and outputs of other neuron in network [75]. The total input to the neuron *i* is given by:

(2) $$\sum_{j\neq i}\frac{v_{j}}{R_{ij}}+I_{i}$$

where *R_ij_* is interconnection weight form neuron *i* to neuron *j*.

Output has two states threshold neurons. In that way, output *v_i_* of neuron *i* is defined by values *v*^0^*_i_* and *v*^1^*_i_*. These two values are noted as 0 and 1. The term *u_i_* represents the threshold of neuron *i*, and output of this neuron is in related to *u_i_* by:

(3) $$\begin{array}{l} if\left(\sum_{j\neq i}\frac{v_{j}}{R_{ij}}+I_{i}<u_{i}\right)\left\{v_{i}=v_{i}^{0}\right\}\\ \text{else}\left\{v_{i}=v_{i}^{1}\right\} \end{array}$$

State equations of the circuit in Figure 11 are described by:

(4) $$C_{i}\frac{du_{i}}{dt}=\underset{j\neq i}{\sum_{j=1}^{N}\frac{v_{j}}{R_{ij}}}-\frac{u_{i}}{R_{i}}+I_{i},i=1,2,\ldots ,N$$

where *R_i_* is an equivalent resistance connected to the cell's capacitor *C_i_*.

If we assume constants *a_i_* sufficiently large, the stability of the network, in Liapunov sense, may be verified by observing the energy function, *E*, describing the state of the network:

(5) $$E=\underset{i\neq j}{-\frac{1}{2}\sum_{i=1}^{N}\sum_{j=1}^{N}\frac{v_{j}v_{i}}{R_{ij}}}-\sum_{i=1}^{N}I_{i}v_{i}$$

The change in energy, due to the change in the state of neuron *i*, is:

(6) $$\frac{\partial E}{\partial v_{i}}=-\sum_{j=1}^{N}\frac{v_{j}}{R_{ij}}-\sum_{i=1}^{N}I_{i}$$

If right side of Equation (6) includes Equation (4), we have:

(7) $$C_{i}\frac{du_{i}}{dt}=-\frac{u_{i}}{R_{i}}-\frac{\partial E}{\partial v_{i}}$$

or, in stable state, when signals are unchangeable: *du_i_ = dt* = 0, one can obtain:

(8) $$\partial E=-\frac{u_{i}}{R_{i}}\partial v_{i}$$

Based on Equation (8), we can conclude that energy change must be either zero or negative. This will be one of system stability conditions.

The architecture of the continuous model that can be implemented using passive and active elements such as resistors, capacitors and Op-Amps is represented in [83]. Based on this network, in [75] the well known TSP optimization problem was solved. Proposed energy function for this solution is:

(9) $$E=\frac{A}{2}\sum_{X}\sum_{i}\sum_{j\neq i}v_{Xi}v_{Xj}+\frac{B}{2}\sum_{i}\sum_{X}\sum_{X\neq Y}v_{Xi}v_{Yi}+\frac{C}{2}{\left(\sum_{X}\sum_{i}v_{Xi}-n\right)}^{2}+\frac{D}{2}\sum_{X}\sum_{Y\neq X}\sum_{i}d_{XY}v_{Xi}(v_{Y,i+1}+v_{Y,i-1})$$

In this way a Hopfield neural network can provide good solution for other NP complete problems [84]. Significant improvements in the neural network algorithm are derived in paper offered by Ali and Kamoun [76]. For *N* routers problem, their computational network uses *N* (*N* − 1) neurons—the diagonal elements in the connection matrix *N* × *N* are removed—and the shortest path from final stable neuron states was found. A suitable energy function is of the form:

(10) $$E=\frac{\mu_{1}}{2}\underset{\left(X,i)\neq (d,s\right)}{\sum_{X}\sum_{i\neq X}C_{Xi}v_{Xi}+}\frac{\mu_{2}}{2}\underset{\left(X,i)\neq (d,s\right)}{\sum_{X}\sum_{i\neq X}\rho_{Xi}v_{Xi}+}\frac{\mu_{5}}{2}(1-v_{ds})+\frac{\mu_{3}}{2}{\sum_{X}\left(\sum_{i\neq X}v_{Xi}-\sum_{i\neq X}v_{iX}\right)}^{2}+\frac{\mu_{4}}{2}\sum_{i}\sum_{X\neq i}v_{Xi}(1-v_{Xi})$$

Coefficients *C_Xi_* are the link costs from router *X* to router *i* and the terms ρ*_Xi_* describe the connection between routers: the value is 1 if routers are not connected, and 0 for connected routers. The terms *μ_i_* are preciously described in [76]. In the same paper authors defined elements of weight matrix as a constant value given by:

(11) $$T_{Xi,Yj}=\mu_{4}\delta_{XY}\delta_{ij}-\mu_{3}(\delta_{XY}+\delta_{ij}-\delta_{jX}-\delta_{iY})$$

In this way, authors suggest final network parameters relation suitable for computational operation as:

(12) $$C_{i}\frac{du_{i}}{dt}=-\frac{u_{i}}{R_{i}}-\frac{\mu_{1}}{2}C_{Xi}(1-\delta_{Xd}\delta_{is})-\frac{\mu_{2}}{2}\rho_{Xi}(1-\delta_{Xd}\delta_{is})-\mu_{3}\sum_{Y\neq X}\left(v_{XY}-v_{YX}\right)+\mu_{3}\sum_{Y\neq X}\left(v_{iY}-v_{Yi}\right)-\frac{\mu_{4}}{2}(1-2v_{Xi})+\frac{\mu_{5}}{2}\delta_{Xd}\delta_{is}$$

Based on (12), our algorithm will try to solve very complex multicriteria optimization [85,86] problem, in order to make WMN routing more effective.

## 5. Route Computation

The main part of routing algorithm refers to the previously mentioned second phase, where the routing algorithm should find the optimal route based on multicriteria decisions [87,88]. Artificial intelligence enables very good results when solving complex problems [75–78]. In our previous papers we have exploited a lot of different neural networks implementations related to routing problems. This routing logic is based on the Hopfield neural network, as it is able to solve one of the most famous problems in graph theory—the TSP problem. For this purpose, we start from the Hopfield neural network algorithm, which has significant improvements realized by Ali and Kamoun [76]. For an *N* nodes problem, their computational network uses *N* (*N* − 1) neurons—the diagonal elements in the connection matrix *N × N* are removed—and the shortest path from final stable neuron states is found. Based on that, a suitable energy function in our algorithm becomes:

(13) $$E=\frac{\mu_{1}}{2}\underset{\left(X,i)\neq (d,s\right)}{\sum_{X}\sum_{i\neq X}[1-(B_{Xi}-L_{Xi})v_{Xi}+}\frac{\mu_{2}}{2}\underset{\left(X,i)\neq (d,s\right)}{\sum_{X}\sum_{i\neq X}\rho_{Xi}v_{Xi}+}\frac{\mu_{3}}{2}{\sum_{X}\left(\sum_{i\neq X}v_{Xi}-\sum_{i\neq X}v_{iX}\right)}^{2}+\frac{\mu_{4}}{2}\sum_{i}\sum_{X\neq i}v_{Xi}(1-v_{Xi})+\frac{\mu_{5}}{2}(1-v_{ds})+\frac{\mu_{6}}{2}\underset{\left(X,i)\neq (d,s\right)}{\sum_{X}\sum_{i\neq X}H_{Xi}v_{Xi}+}\frac{\mu_{7}}{2}\underset{\left(X,i)\neq (d,s\right)}{\sum_{X}\sum_{i\neq X}D_{Xi}v_{Xi}}$$

Coefficients *B_Xi_* denote the bandwidth of link from node *X* to node *i* and the terms ρ*_Xi_* describe the connections between nodes: the value is 1 if nodes are not connected, and 0 for connected nodes. In the same way coefficients *L_Xi_* describe traffic load of link from node *X* to node *i*, *D_Xi_* correspond to the link delays and *H_Xi_* represent modified coefficients *B_Xi_* in order to use hop count metric. The term (*B_Xi_* − *L_Xi_*) relates to available link capacity and the routing protocol has to find a path with as much as possible free space in link capacity. Since the neural network is designed to minimize the energy function, actual term in energy function has to be in the form [1−(*B*_Xi_ − *L*_Xi_)] [85].

The constant *μ*_1_ minimizes available bandwidth on the link, μ_6_ minimizes the number of hops and *μ*_7_ minimizes total delay. Constants *μ*_2_, *μ*_3_, *μ*_4_ and *μ*_5_ have the same meaning as in original Ali and Kamoun paper [76].

By default, constant *μ*_6_ and *μ*_7_ are set to 0. On this way hop count and delay are not included in the metrics. Basic metrics is realized by link bandwidth and link load. In this way, blocking probability should be minimized.

Starting from synaptic conductances given by Equation (11) and from the bias currents *I_Xi_* as:

(14) $$I_{Xi}=-\frac{\mu_{1}}{2}[1-(B_{Xi}-L_{Xi})(1-\delta_{Xd}\delta_{is})-\frac{\mu_{2}}{2}\rho_{Xi}(1-\delta_{Xd}\delta_{is})-\frac{\mu_{4}}{2}+\frac{\mu_{5}}{2}\delta_{Xd}\delta_{is}-\frac{\mu_{6}}{2}H_{Xi}(1-\delta_{Xd}\delta_{is})-\frac{\mu_{7}}{2}D_{Xi}(1-\delta_{Xd}\delta_{is})$$

we created Hopfield neural network model suitable for real time processes.

The Hopfield neural network model is realized as a function within the Matlab code, and is activated every time when routing protocol needs some new route. As the function arguments the number of nodes, bandwidth values and load parameters on the links (and optionally delays) are used. As a result, we will get output matrix **V**. Values *V_Xi_* are 1 (if link between node *X* and node *i* is included in final optimal route) or 0 in other case [85]. Based on this route, all routing tables should be changed for a particular user. For this purpose one new mobile agent is created and sent through this route to bring this information to routing tables in associated nodes.

## 6. Results

In this section we present the results of the simulation which have been performed in the Matlab environment. Although the Matlab offers very rich toolboxes, among others the Neural Network Toolbox, we used the Matlab only as a programming tool and created our own neural network model, as described in Section 5, since in this way we are able to control the neural network dynamically, assuming the actual traffic conditions.

### 6.1. Design Network Environment

**Figure 13.**
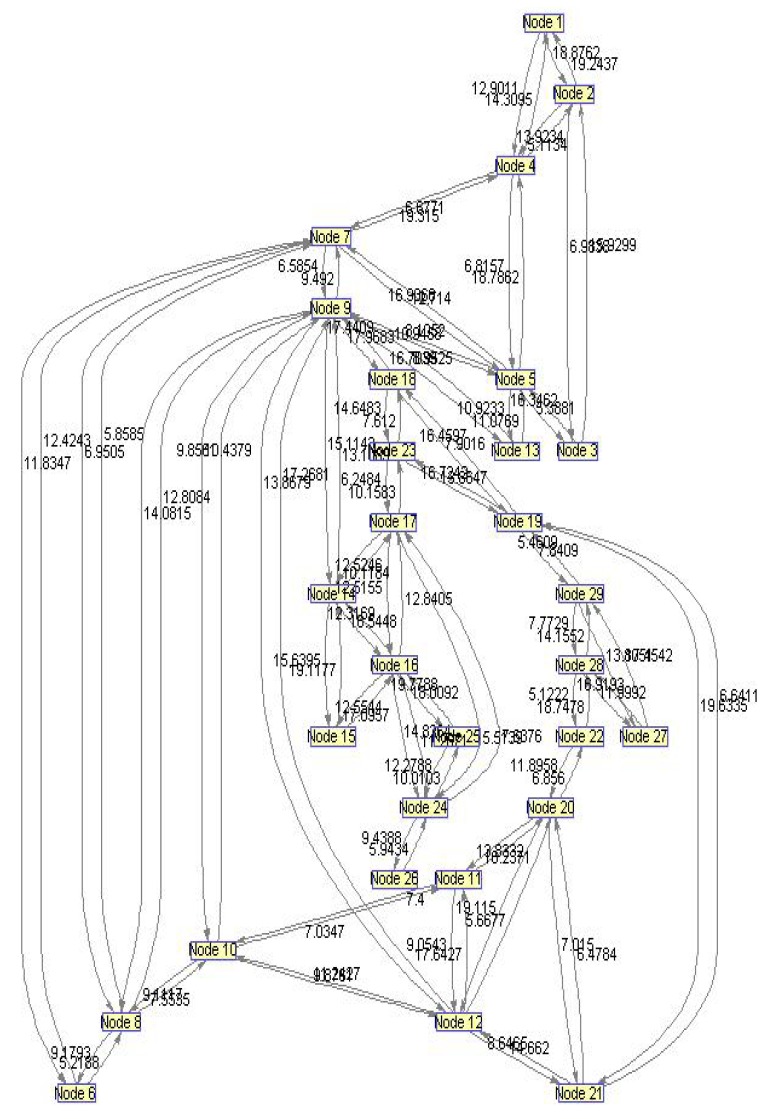
Network topology used for simulation.

In order to evaluate the influence of the following parameters: traffic load, network size, network delay, average link usage, intensive simulations have been done. We created separate code functions to make the network environment. This code generates random connectivity of *N* nodes which belong to one network. For this purpose, we defined network based on *N* = 29 nodes and 48 bidirectional links, shown in Figure 13.

**Figure 14.**
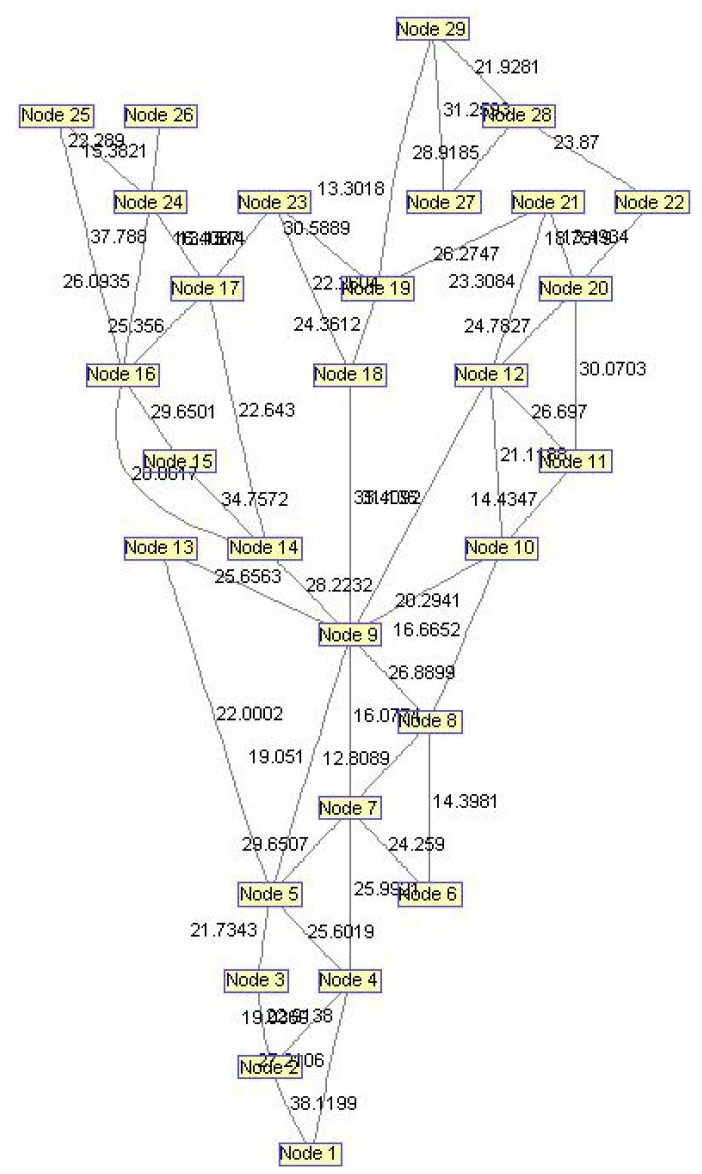
Network topology—simplified preview.

By applying the Bioinformatics toolbox from Matlab 2008 on the network from Figure 13, we obtained the unidirectional graph in Biograph viewer, as depicted in Figure 14. Based on a real network environment we assumed that bandwidth of every wireless link is between 5 Mbps and 40 Mbps. Every link is described by bandwidth parameters randomly chosen from supposed interval. We have also assumed that every new user connected to network has the bandwidth, randomly chosen from 1–20 Mbps.

We have performed intensive simulations for network topology shown in Figure 13. In every simulation, network parameters were randomly chosen: the number of new users, nodes through which new users are connected to the network, time of connection and sets of nodes which are gateways. For all of these simulations, the main problem was addressed to the traffic density. We suppose that the size of average data packet is 2 kB. Traffic is randomly generated for every new user. Simulation model make reservation for this traffic through all links included in the route path. This reservation is expressed through additional value of the link parameters *L_Xi_*. For testing the proposed routing algorithm in highly dynamic network environment, we supposed that during one simulation every new user needs more traffic than the previous one. Due to that, with every new user the routing process becomes more complex. One simulation interval implies fixed network topology with randomly distributed gateways and load for every new user. One interval is limited to 10 min. During this time, *M* of the new users should be connected to the network. Initializations of these connections are randomly distributed but the time period between two connections is larger than 2 s. Load for every user is randomly generated for every new interval. For making different sets of network performances we simulated a lot of specific intervals. For every interval we generated different load. We used factor *k*, as a measure of traffic load. It means, load is given as:

(15) load=rand(N)×k

**Figure 15.**
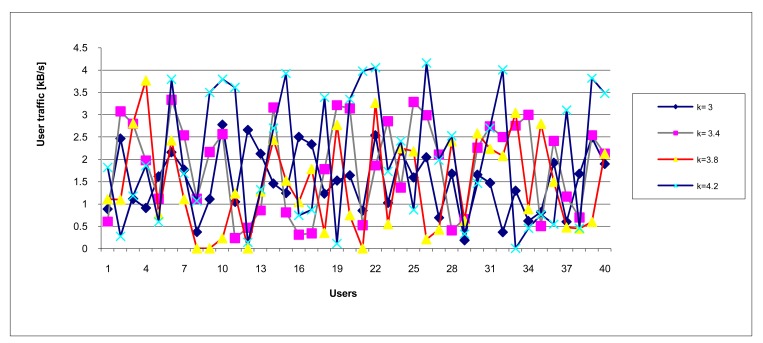
Traffic load distribution (lower density, *k* < 4.5).

**Figure 16.**
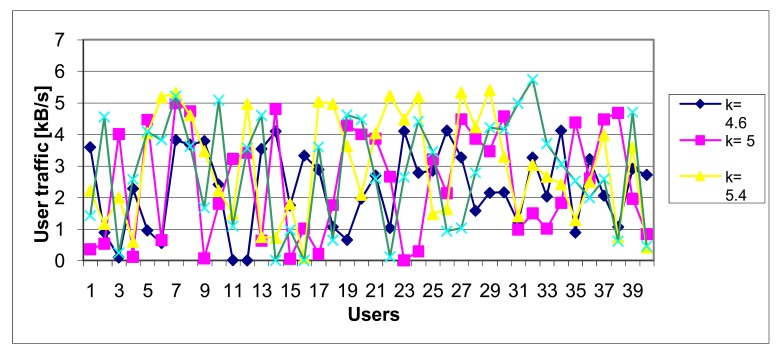
Traffic load distribution (higher density, *k* > 4.5).

For various simulation we used *k* = [2.5; 2.8; 3.0; 3.2; 3.4; 3.6; 3.8; 4.0; 4.2; 4.4; 4.6; 4.8; 5.0; 5.4; 5.6; 6.0; 7.0]. Some of diagrams illustrating traffic load distribution are depicted in Figure 15 and Figure 16.

The third parameter associated to links is the delay. This parameter is also randomly generated and should indicate transmission delay over the link. The described network model is used for all simulations. Dynamic changes of input parameters offer us the possibility to use this model for different and real time network simulations.

### 6.2. Simulations

Metrics used for performances analysis of the proposed routing protocol are presented here. For this purpose we compare the proposed algorithm with two others: AODV [18,36,37] and OSPF [24]. AODV is one of the most used protocols in the literature for WMNs. Many authors have used this protocol to compare either its original form or many of its modifications. Our proposed algorithm is a hybrid one, but it is closer to the reactive than the proactive [3] type. If we know that AODV represents a reactive protocol, it is obvious that we should compare with it. On the other hand, our protocol is a link state protocol which includes bandwidth and load in its final decisions. There are many link state protocols described in the literature [1,9,14,17,24,28]. We considered OSPF as a well known and most used protocol addressed to wired networks. From the standpoint of defined metrics a more appropriate comparison would be with the Enhanced Interior Gateway Routing Protocol (EIGRP). However, EIGRP is a proprietary Cisco routing protocol opposite to OSPF. From that reason many authors have a lot of different modifications and possible implementations of OSPF. All these reasons are of high importance when selecting AODV and OSPF protocols. We use the following metrics to compare the performance of the three routing protocols:

1. Packet delivery ratio,
2. Throughput,
3. End-to-end delay,
4. Average hop count,
5. Blocking iterations.

#### 6.2.1. Packet Delivery Ratio

**Figure 17.**
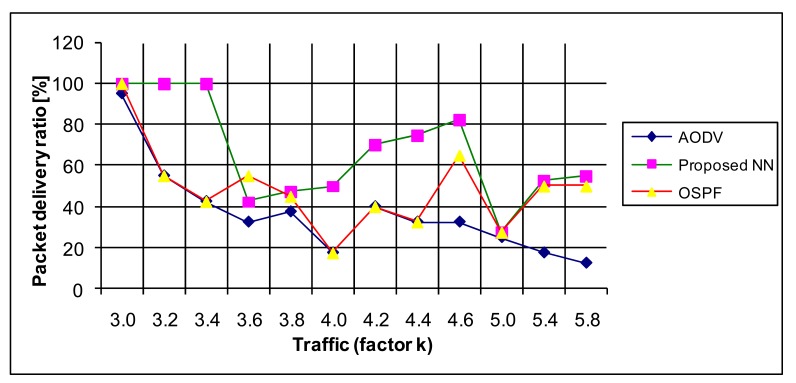
The performance of the routing protocols—PDR.

The packet delivery ratio (PDR) is defined as the ratio between the packets that are received and the number of packets sent. This is one of the most used metrics for protocol comparison. In our simulations the proposed NN model exhibits the best performance, then the OSPF, as depicted in Figure 17.

#### 6.2.2. Throughput

The throughput between two nodes is expressed as the number of bytes delivered per unit of time. Formally:

(16) Throughput=Total bytes received/Total time

**Figure 18.**
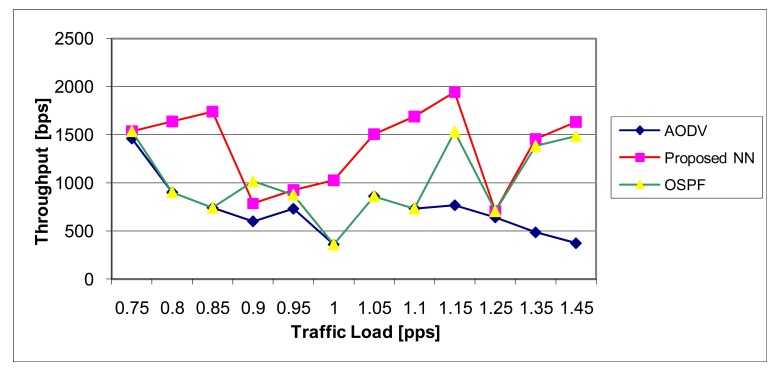
The performance of the routing protocols—Throughput.

The throughput (measured by bytes per seconds) we have calculated as a function of the traffic load (expressed as the number of equal-sized packets per second), for different routing protocols, and results are depicted in Figure 18. The proposed NN algorithm gives significantly better results than AODV and OSPF. This is based on fact that the proposed algorithm is tailored for finding the optimal routes and thus increasing the number of packets which reach the destination.

#### 6.2.3. End-to-End Delay

The end-to-end packet delay is calculated as the time interval between the time instant when the packet is generated and is ready for the transmission, and the moment when it reached the destination node.

**Figure 19.**
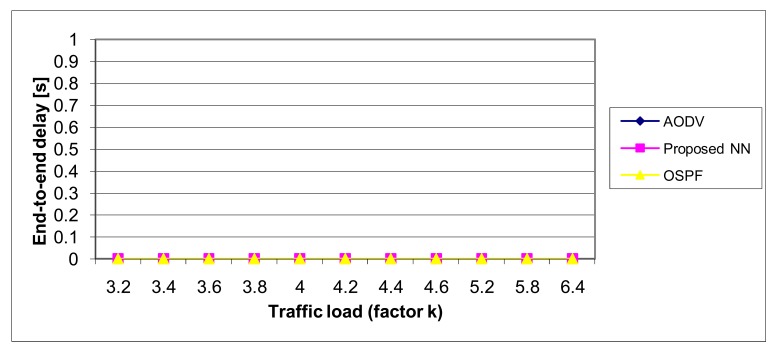
The performance of the routing protocols—End-to-end delay.

Simulation results, depicted in Figure 19, show that the OSPF is better than the proposed NN method (for about 20%) while AODV is significantly worse, but note that we have the full benefits of neural networks can be achieved only through the hardware implementation, when the parallel work of all neurons is possible. Instead of that, we are using simulation, meaning our model was working in sequential mode which is unnatural for neural networks.

#### 6.2.4. Average Hop Count

**Figure 20.**
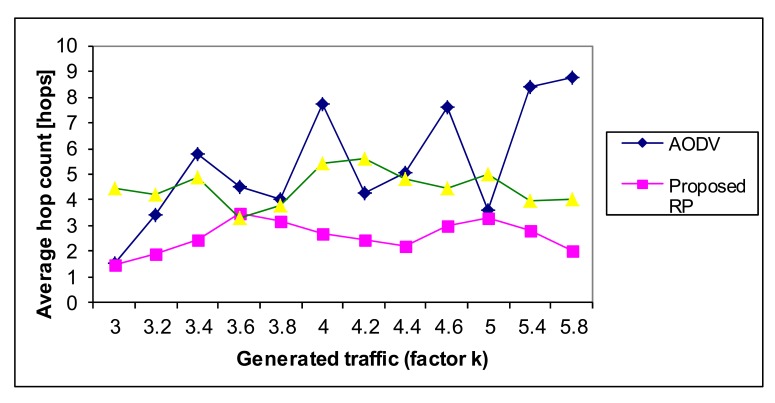
The performance of the routing protocols—Average hop count.

Average hop count (measured as the number of hops) is an average number of used particular links in the route. This metric indicates how good the routing protocol is regarding the network resources. Sometimes, routes with minimal hop count need not be optimal from the point of all the observed network parameters. However, as less links are involved in the final route, the selection is better. In our case, the proposed NN algorithm has the smallest average hop count (Figure 20), due to the fact that the NN can find the Pareto optimal route and then the network resources are optimally used.

#### 6.2.5. Blocking Iterations

**Figure 21.**
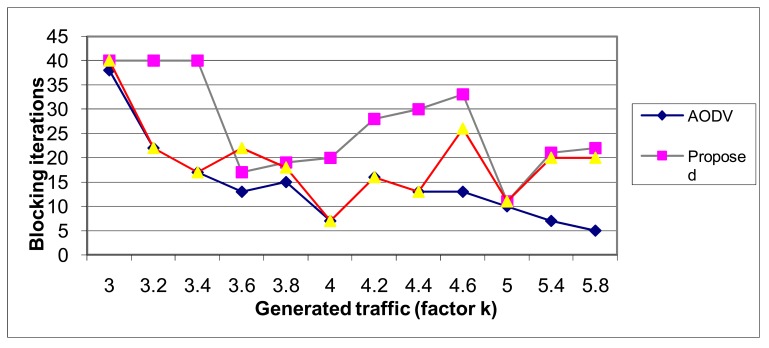
The performance of the routing protocols—Blocking iterations.

This metrics can be expressed as the number of iterations before blocking the traffic particular user and is often used in optical networks or in multi channel networks blocking probability quantification. If we suppose that new users generate the same traffic all the time during connection (independent of sending or receiving processes) the routing algorithm should reserve enough bandwidth on every link on the route. Depending on the routing algorithm and its ability to use available links in an optimal way traffic jams can be avoided in more or less extent. The neural network method exhibits again the best results, as depicted in Figure 21, and regarding the optimization of network resources. Moreover, t, as depicted in Figure 21.

After analyzing all the presented results we can conclude that the proposed routing algorithm based on the artificial neural network could be efficiently used in wireless mesh networks. In more than 96% cases of intensive simulations under different and randomly changed network and traffic conditions, the proposed protocol was better than AODV and OSPF. It must be stressed that the NN simulation was performed by digital computer, meaning, by working in sequential mode which is unnatural for neural networks. It is well-known that neural networks exhibit significantly better results in their hardware realization when the natural parallel mode of operation is possible, producing dramatically shorter execution times, but in our research we concentrated on the architecture and organization of a new routing protocol which includes the artificial intelligence in the routing process.

## 7. Conclusions

A lot of implementations of wireless mesh networks and their significant advantages over competing technologies need every day improvements from the point of QoS. The main part of the desired quality and network stability is the routing protocol. In this paper, a new hybrid routing protocol for wireless mesh networks is presented. We have created new routing metrics based on multicriteria optimization. We have observed several network parameters in order to provide optimized usage of network resources and network stability. For this purpose we have started with Hopfield neural network and suggested its modification addressed to the improvements of the routing performances in real conditions. Intensive simulations confirm that the use of artificial intelligence can be very efficient in routing, even in the case of dynamical environment.

The proposed routing protocol is compared with two well known routing protocols. We used several metrics for describing the performances of the routing algorithms. It is shown that the proposed routing protocol has better or the same performance in all metrics except one, even though the neural network is simulated by a digital computer. Our protocol is scalable and it is adapted for dynamic network topology and real network environments.

We believe that the performance of the proposed routing protocol can be further improved if we use multi-radio or multi-channel routing. We will explore this option in our future work. Also, hardware realization of the neural network will significantly improve the execution time for the optimization and decision processes.
